# Gallbladder Mixed Neuroendocrine–Non-Neuroendocrine Neoplasm Consisting of Adenocarcinoma and Neuroendocrine Tumor G2 Diagnosed after Surgery for Acute Cholecystitis: A Case Report and Exome Analysis

**DOI:** 10.70352/scrj.cr.24-0097

**Published:** 2025-06-07

**Authors:** Takaomi Seki, Hideki Suzuki, Yoshiyasu Takayama, Yohei Morishita, Kosuke Taniguchi, Reika Kawabata-Iwakawa, Takehiko Yokobori, Hayato Ikota, Takahiro Shirakura, Kenichiro Araki, Kenichiro Hata, Ken Shirabe

**Affiliations:** 1 Department of Surgery, Isesaki Municipal Hospital, Isesaki, Gunma, Japan; 2 Department of General Surgical Science, Division of Hepatobiliary and Pancreatic Surgery, Graduate School of Medicine, Gunma University, Maebashi, Gunma, Japan; 3 Department of Pathology, Isesaki Municipal Hospital, Isesaki, Gunma, Japan; 4 Laboratory for Analytical Instruments, Education and Research Support Centre, Graduate School of Medicine, Gunma University, Maebashi, Gunma, Japan; 5 Department of Human Molecular Genetics, Graduate School of Medicine, Gunma University, Maebashi, Gunma, Japan; 6 Division of Integrated Oncology Research, Gunma University Initiative for Advanced Research, Gunma University, Maebashi, Gunma, Japan; 7 Department of Diagnostic Pathology, Gunma University Hospital, Maebashi, Gunma, Japan

**Keywords:** mixed neuroendocrine–non-neuroendocrine neoplasms (MiNENs), gallbladder cancer, acute cholecystitis

## Abstract

**INTRODUCTION:**

Among neuroendocrine neoplasms (NENs), non-neuroendocrine and NEN components may rarely coexist, which are referred to as mixed neuroendocrine–non-NENs (MiNENs). Most gallbladder MiNENs are progressive and associated with neuroendocrine carcinoma (NEC), but rarely with neuroendocrine tumor (NET) as a component. To our knowledge, there are 4 reported cases of mixed gallbladder tumors with NET as a component. From the genetic analysis of MiNENs consisting of NEC, MiNEN is believed to have a common origin, as each tumor component shares a common *TP53* mutation. Our case is an extremely rare reported case of a mixed gallbladder tumor with a NET component as a MiNEN, and the first reported case of whole-exome analysis performed on a resected specimen.

**CASE PRESENTATION:**

A 77-year-old woman presented to our hospital with epigastric pain. An emergency laparoscopic cholecystectomy was performed with a diagnosis of acute gallstone cholecystitis. Pathological examination revealed gallbladder MiNEN (adenocarcinoma + NET G2). Additional surgery was performed, but no residual tumor was found. The patient has been recurrence-free for 36 months after surgery without adjuvant therapy. The origin of the tumor was examined. Macroscopically, adenocarcinoma cells were present on both sides of the NET, while microscopically, some adenocarcinoma cells were positive for neuroendocrine markers (synaptophysin and chromogranin A). Staining for p53 showed wild-type staining with scattered, weakly expressing cells in both tumors. Subsequently, we performed whole-exome sequencing of each tumor component. The results showed that each tumor component shared *TP53* c.1015G>T (p.Glu339Ter), *ERBB3* c.889G>A (p.Asp297Asn), and *CDKN2A* c.416G>A (p.Gly139Asp) mutations, suggesting that the adenocarcinoma might have differentiated into NET G2.

**CONCLUSIONS:**

In this case report, the tumors shared a common genetic mutation, suggesting that MiNENs with NET components may share a common origin. Furthermore, the NEN component of MiNEN occurring in the gallbladder was associated with a *TP53* mutation, despite the low frequency of *TP53* mutations in normal NETs.

## Abbreviations

CA19-9
: carbohydrate antigen 19-9
CEA
: carcinoembryonic antigen
FDG
: F-18 fluorodeoxyglucose
FFPE
: formalin-fixed paraffin-embedded
MiNEN
: mixed neuroendocrine–non-neuroendocrine neoplasms
NEC
: neuroendocrine carcinoma
NEN
: neuroendocrine neoplasm
NET
: neuroendocrine tumor
PE
: paired-end
SEER
: Surveillance, Epidemiology, and End Results
WES
: whole-exome analysis

## INTRODUCTION

NENs are rare. Gallbladder NENs are even rarer, accounting for only 0.5% of all NENs and 2% of all gallbladder tumors.^1)^ Non-neuroendocrine and NEN components may coexist in a condition referred to as MiNENs. MiNEN is a relatively new disease concept, defined in the 2019 World Health Organization (WHO) classification as a tumor characterized by the coexistence of neuroendocrine and non-NETs in ≥30% of the cases.^2)^ MiNEN is also rare, and many aspects remain unknown. In the SEER database, the frequency of biliary NENs by site was 51% in the gallbladder, 35% in the papilla, and 13% in the extrahepatic bile duct. The histological types were reported as 29% NET, 50% NEC, and 20% MiNEN. Gallbladder MiNENs account for 22% of gallbladder NENs.^3)^ Most gallbladder MiNENs are progressive and associated with NEC, but rarely with NET as a component. To our knowledge, there are only 4 reported cases of mixed tumors with NET as a component,^4–7)^ and only 1 case was reported after the MiNEN diagnostic criteria were established.^7)^

Large-scale genetic analysis of gastrointestinal NENs has led us to believe that NETs and NECs are genomically distinct.^8)^ *TP53* mutations are found frequently in NECs but rarely in NETs. On the other hand, MiNEN is believed to have a common origin, as each tumor component shares a common *TP53* mutation.^9)^

Herein, we present a case of MiNEN consisting of adenocarcinoma and NET G2. Finally, since there are no reports of genomic analysis of MiNEN composed of NET G2, WES was performed on resected specimens. Our case is an extremely rare reported case of a mixed gallbladder tumor with a NET component in MiNEN, and the first reported case of WES performed on a resected specimen.

## CASE PRESENTATION

**Fig. 1:**
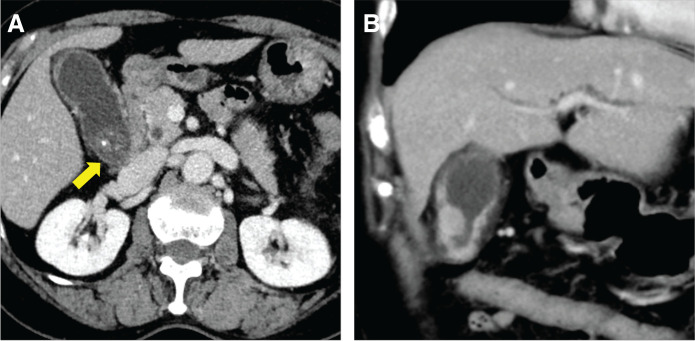
Computed tomography findings. (**A**) Multiple incarcerated gallstones (yellow arrow) are observed. (**B**) Swelling of the gallbladder and edematous circumferential thickening of the gallbladder wall are observed.

**Fig. 2:**
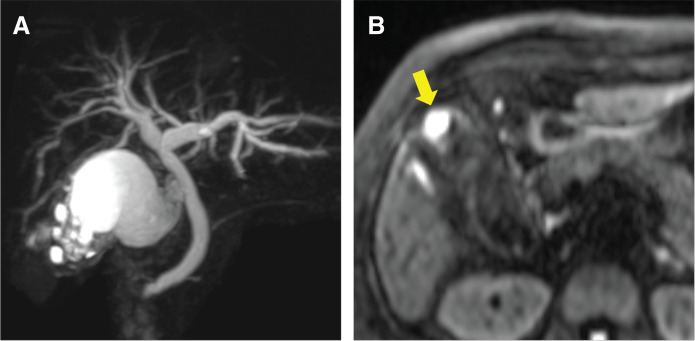
Magnetic resonance imaging findings. (**A**) A Rokitansky–Aschoff sinus is observed, and gallbladder adenomyomatosis is suspected. (**B**) Diffusion-weighted imaging shows nodular thickening of the fundus of the gallbladder with restricted diffusion (yellow arrow).

**Fig. 3:**
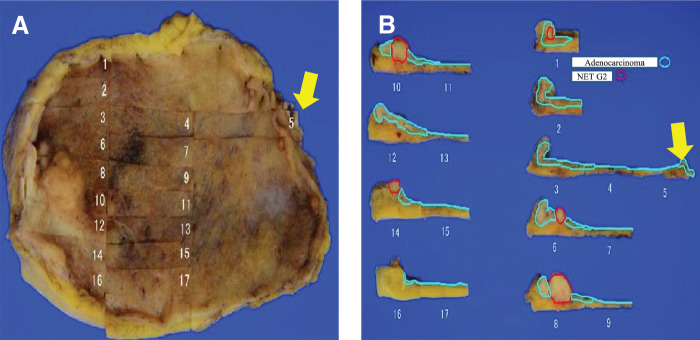
Macroscopic findings of the resected specimen. (**A**, **B**) Adenocarcinoma is in close proximity to the edge of the cystic duct (yellow arrow). NET, neuroendocrine tumor

**Fig. 4:**
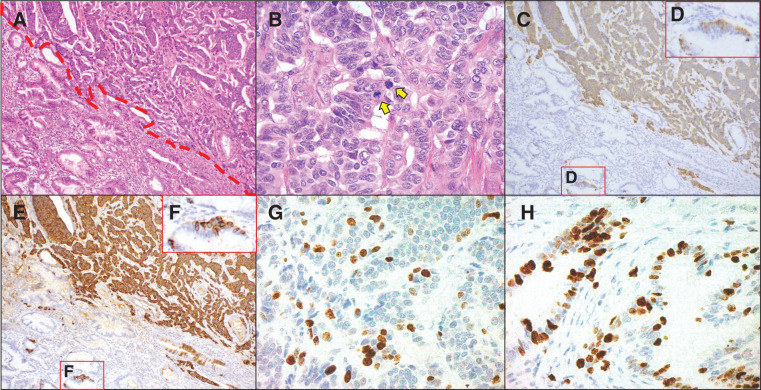
Histopathologic and immunohistochemical findings. (**A**) The borderline between adenocarcinoma and neuroendocrine tumor at low magnification (H&E staining, ×100). The red dotted line is the border; above the line is the neuroendocrine tumor, and below the line is adenocarcinoma. (**B**) Areas with multiple mitotic figures in the neuroendocrine tumor. Tumor cells with a high nucleo-cytoplasmic ratio and rough chromatin form nests, cords, and ribbons and invade. Two mitotic figures are observed in a high-power field (arrow) (H&E staining, ×400). (**C**, **D**) The neuroendocrine tumor and some of the adenocarcinoma cells are positive for chromogranin A (**C**: ×100; **D**: ×400). (**E**, **F**) The neuroendocrine tumor and some of the adenocarcinoma cells are positive for synaptophysin (**E**: ×100; **F**: ×400). (**G**) Ki-67 staining of the NET G2. The percentage of cells expressing Ki-67 is 70/500 (positive cells/total cells), 14% (×400). (**H**) Ki-67 staining of adenocarcinoma. The percentage of cells expressing Ki-67 is 230/500 (positive cells/total cells), 46% (×400). H&E, hematoxylin and eosin; NET, neuroendocrine tumor

A 77-year-old woman presented to our hospital with a gradual worsening of epigastric pain. She had no comorbidities, had been spayed, but had no history of cancer and no blood relatives with cancer. Blood tests showed signs of inflammation and hepatobiliary enzyme abnormalities, but no abnormalities in tumor markers such as CEA and CA19-9. Physical examination revealed right hypochondriac tenderness and Murphy’s sign. Enhanced computed tomography (CT) showed multiple gallstones (**Fig. 1A**), swelling of the gallbladder, and edematous thickening of the gallbladder wall (**Fig. 1B**). Magnetic resonance imaging (MRI) revealed a Rokitansky–Aschoff sinus, and gallbladder adenomyomatosis was suspected (**Fig. 2A**). In contrast, diffusion-weighted imaging revealed nodular thickening of the fundus of the gallbladder, which showed restricted diffusion (**Fig. 2B**). The patient was diagnosed with acute cholecystitis, and the wall thickening of the fundus of the gallbladder was potentially malignant. On the other hand, the patient’s abdominal symptoms were severe, and peritonitis was suspected. Emergency laparoscopic cholecystectomy was performed for rapid symptomatic improvement and diagnostic treatment. Additionally, the gallbladder was carefully removed to prevent bile leakage because the risk of malignancy could not be ruled out based on the preoperative diagnosis. The intraoperative findings included severe cholecystitis with adhesions of white moss and omentum in the gallbladder. Surgery was completed without gallbladder perforation. The postoperative course was uneventful, and the patient was discharged promptly. However, the postoperative pathological diagnosis was MiNEN, comprising adenocarcinoma and NET G2. The neuroendocrine component of the tumor accounted for 30%–40% of the total tumor. The deepest part of the tumor was the subserous layer (pT2b); furthermore, the tumor was close to the margin of the cystic duct (**Fig. 3**). **Figure 4A** shows the borders of each tumor, in which the epithelial component was a moderately to well-differentiated adenocarcinoma and the neuroendocrine component was well differentiated. Tumor cells with a high nucleo-cytoplasmic ratio and rough chromatin formed nests, cords, and ribbons and invaded (**Fig. 4B**). NET and some of the adenocarcinoma cells were positive for chromogranin A (**Fig. 4C** and **4D**) and synaptophysin (**Fig. 4E** and **4F**), which are typical neural markers. No diffuse proliferation or necrosis was observed. The number of mitotic figures was 5 per 10 high-power fields, and the proportion of cells expressing Ki-67 was 70/500 (positive cells/total cells), or 14% (**Fig. 4G**), indicating that the proliferative potential was not very high. On the other hand, the percentage of cells expressing Ki-67 in adenocarcinoma was 230/500, or 46% (**Fig. 4H**), suggesting that adenocarcinoma had a higher proliferation ability than NET. These findings are consistent with NET rather than NEC. Because there was a possibility of residual tumor or distant metastasis, FDG positron emission tomography (FDG-PET) was performed to determine the treatment strategy required for curative treatment, but no abnormal accumulation of FDG was observed. Blood tests did not reveal any elevation in CEA, CA19-9, or neuron-specific enolase. Since we judged that lymph node and distant metastases, such as liver metastasis, were unlikely and that radical resection was possible with additional local resection, we performed surgery. The surgical procedures included hepatectomy of segments 4b and 5, extrahepatic bile duct resection, choledochojejunostomy, and lymph node dissection. First, we carefully checked the abdominal cavity, but no peritoneal dissemination or liver metastasis was found. Pathological examination revealed no residual tumor in the resected specimen, and intraoperative peritoneal washing cytology revealed no malignant findings. The final diagnosis was gallbladder MiNEN consisting of adenocarcinoma and NET G2 (classified as T2bN0M0 stage II according to the 8th edition of the Union for International Cancer Control staging system). The postoperative course was uneventful. The patient did not receive postoperative adjuvant therapy at her request and was recurrence-free 36 months after the surgery.

**Fig. 5:**
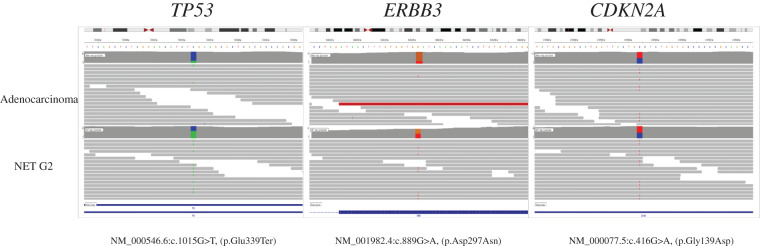
Illustration of the *TP53, ERBB3*, and *CDK2NA* gene alterations in each tumor component. WES data, visualized using Integrative Genomics Viewer software, reveal identical *TP53* c.1015G>T (p.Glu339Ter), *ERBB3* c.889G>A (p.Asp297Asn), and *CDKN2A* c.416G>A (p.Gly139Asp) mutations in each tumor component. NET, neuroendocrine tumor; WES, whole-exome sequencing

Each tumor was distinct, and there was a possibility of collision tumors. Therefore, we were interested in the origin of the tumors. In MiNEN, each tumor component often has a common *TP53* mutation. Therefore, WES was performed for each tumor component. Each tumor component was macrodissected from FFPE tissue, avoiding sampling from the tumor borders to prevent contamination. DNA extraction was performed using the GeneRead DNA FFPE kit (Qiagen, Hilden, Germany) following the manufacturer’s protocol. SureSelect Human All Exon V6 kit (Agilent Technologies, Santa Clara, CA, USA) was used to construct an Illumina PE library. PE sequencing (2 × 150 bp) was performed using the Illumina NovaSeq 6000 platform (Illumina, San Diego, CA, USA). We performed alignment, mapping, and annotation based on a previous report.^10)^ Both tumor components were found to share mutations in *TP53* c.1015G>T (p.Glu339Ter), *ERBB3* c.889G>A (p.Asp297Asn), and *CDKN2A* c.416G>A (p.Gly139Asp) (**Fig. 5**). Similar to previous reports, the pathogenic variant of *TP53* was identified in each tumor component of MiNEN, suggesting a common origin in the present case.

## DISCUSSION

MiNEN is a relatively new disease concept defined in the 2019 WHO classification as a tumor characterized by the coexistence of neuroendocrine and non-NET in ≥30% of the cases.^2)^ Among gallbladder NENs, the proportion of gallbladder MiNENs is reported to be 22% in the SEER database. On the other hand, Wang et al. reported that MiNEN accounted for 9 out of 13 (approximately 69%) gallbladder NENs.^7)^ The exact frequency is unknown because of the small number of cases. On the other hand, the proportion of pancreaticobiliary MiNENs has been reported to be 3.2%.^9)^ According to several reviews,^11,12)^ MiNEN is usually more common in men, but gallbladder MiNEN is more common in women. Most patients complain of pain and discomfort in the upper right or upper abdomen, which are complicated by cholelithiasis. Imaging studies, such as ultrasonography, CT, MRI, FDG-PET, and somatostatin receptor scintigraphy, can provide useful information. However, the imaging findings of gallbladder NETs are nonspecific, making it difficult to differentiate gallbladder NETs from gallbladder adenocarcinomas based on imaging findings. MiNEN is a broad disease concept and has been proposed to be classified into 3 grades according to histologic type for treatment selection and prognostic prediction.^9)^ Regarding prognosis, the median survival duration after surgery for gallbladder MiNEN is reported to be 11.5−36 months.^7,12)^

Most of the neuroendocrine components of gallbladder MiNEN are NECs. However, in our case, the gallbladder MiNEN consisted of NET G2. To our knowledge, 5 cases, including ours, have been reported, with 4 cases having combined adenocarcinoma and NET G2.^4–7)^ These cases are summarized in **Table 1**. To summarize the characteristics, the tumor was located at the fundus of the gallbladder in all 4 reported cases. The median tumor size was 45 mm (range, 20–82 mm), which is relatively large and easily recognized as a tumor. Although the details are unknown, 1 case of death has been reported,^7)^ whereas the other cases have progressed without recurrence.

**Table 1:** Reported cases of gallbladder mixed neuroendocrine–non-neuroendocrine neoplasm consisting of neuroendocrine tumor G2

Case	Author	Year	Age/gender	Tumor site/ size (mm)	Surgery	Epithelial component	Ki-67 (%)	Mitoses (/10 high power fields)	Outcome
1	Harada et al.^4)^	2012	70/F	Unknown/45	Unknown	Well-differentiated adenocarcinoma	0.5	4	Unknown
2	Shintaku et al.^5)^	2013	80/M	Fundus/82	Cholecystectomy, dissection of regional lymph nodes	Tubular adenocarcinoma, squamous cell carcinoma, osteosarcoma	18.7	6.2	Alive (8 months) with no recurrence
3	Azad et al.^6)^	2015	62/M	Fundus/20	Cholecystectomy, hepatectomy of segments 4b and 5, dissection of regional lymph nodes	Moderately differentiated adenocarcinoma	15	Unknown	Alive (24 months) with no recurrence
4	Wang et al.^7)^	2021	75/F	Fundus/70	Cholecystectomy, partial liver resection, dissection of regional lymph nodes	Well-differentiated adenocarcinoma	10	10	Death (10 months)
5	Our case	—	77/F	Fundus/45	Laparoscopic cholecystectomy → hepatectomy of segments 4b and 5, dissection of regional lymph nodes	Well- to moderately differentiated adenocarcinoma	14	5	Alive (36 months) with no recurrence

F: female; M: male

**Fig. 6:**
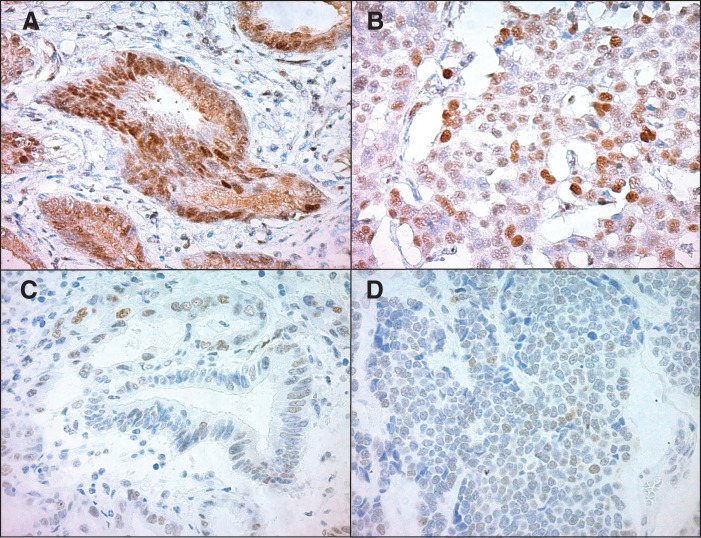
Immunohistochemical findings. (**A**) Rb1 staining in adenocarcinoma. Rb1 expression is observed in the nuclei of tumor cells (×400). (**B**) Rb1 staining in NET G2. Rb1 expression is observed in the nuclei of tumor cells (×400). (**C**) p53 staining in adenocarcinoma. Tumor cells show weak and scattered p53 expression (usual pattern) (×400). (**D**) p53 staining in NET G2. Similar to adenocarcinoma, the tumor cells show weak and scattered p53 expression (usual pattern) (×400). NET, neuroendocrine tumor

MiNEN is believed to have a common origin, as each tumor component often shares a common *TP53* mutation.^9)^ There are 5 cases of genetic analysis of gallbladder MiNEN, of which 4 had the same *TP53* mutation in each of the gallbladder MiNENs.^13−15)^ These cases are summarized in **Table 2**. Large-scale genetic analysis of gastrointestinal NEN has led us to believe that NET and NEC are genomically distinct.^8)^ *TP53* mutations are rare in well-differentiated NETs, whereas missense mutations in *TP53* are a common mutation pattern in NECs, leading to overexpression of the p53 protein. Based on these results, *TP53* mutations and the associated overexpression of p53 protein are considered strong molecular markers for distinguishing NETs from NECs.^16)^ Therefore, we decided to perform immunohistochemical staining and genetic analysis in the present case to determine whether MiNEN composed of NET shares the *TP53* mutation. First, we stained for Rb1 to confirm the diagnosis of NET G2. Rb1 expression was found in both adenocarcinoma (**Fig. 6A**) and NEN (**Fig. 6B**), with no loss of Rb, suggesting that the neuroendocrine component was an NET, not an NEC. Staining for p53 revealed scattered, weakly expressing cells in both adenocarcinoma (**Fig. 6C**) and NET (**Fig. 6D**) that showed a normal rather than a null expression pattern.

**Table 2:** Reported cases of mixed neuroendocrine and non-neuroendocrine neoplasia of the gallbladder with genetic analysis

Case	Author	Year	Component	Common gene mutation
1	Sta.Ines et al.^13)^	2019	AC, NEC	*TP53* c.273G>A (p. Trp91Ter)
2	Sciarra et al.^14)^	2020	AC, NEC, ICPN	*TP53* c.700T>C (p. Tyr234His)
3	de Bitter et al.^15)^	2021	AC, NEC, ICPN	*TP53*, *BRCA2*
4	de Bitter et al.^15)^	2021	AC, NEC	*TP53*, *CTNNB1*, *FANCA*
5	de Bitter et al.^15)^	2021	AC, NEC	None *Different TP53 mutations in AC and NEC
6	Our case	–	AC, NET G2	*TP53* c.1015G>T (p.Glu339Ter), *ERBB3* c.889G>A (p.Asp297Asn), *CDKN2A* c.416G>A (p.Gly139Asp)

AC, adenocarcinoma; ICPN, intracystic papillary neoplasms; NEC, neuroendocrine carcinoma; NET, neuroendocrine tumor

Subsequently, WES analysis was performed on each tumor component and identified *TP53*, *ERBB3*, and *CDKN2A* mutations in both tumor components. The germline *TP53* mutation (NM_000546.6:c.1015G>T [p.Glu339Ter]) identified in this case is a mutation previously reported in Li–Fraumeni syndrome. In previous reports,^13,14)^ a *TP53* mutation similar to that reported in Li–Fraumeni syndrome was detected; however, it was not considered a germline mutation but a somatic mutation. In addition, since the patient in the present case had no personal or family history of cancer, the *TP53* mutation is more likely to be a somatic mutation. Both *ERBB3* and *CDKN2A* variants have been reported as somatic mutations in the Catalogue Of Somatic Mutations In Cancer database (https://cancer.sanger.ac.uk/cosmic). *CDKN2A* is known as a specific driver gene for biliary tract cancer,^17)^ but the variant (c.416G>A [p.Gly139Asp]) detected in this study was reported to be of unknown pathogenic significance. On the other hand, *ERBB3* is known as a driver gene specific to gallbladder cancer.^18)^ The variant detected this time (c.889G>A [p.Asp297Asn]) has been reported as a somatic mutation in uterine cancer, gastric cancer, and so on. Regarding the frequency of germline mutations, the Genome Aggregation Database (https://gnomad.broadinstitute.org/) shows that *CDKN2A* is rare and *ERBB3* has not been reported. Since the *ERBB3* variant has a Combined Annotation Dependent Depletion phred score of 25 or higher, it is predicted to affect protein function and is therefore considered to be of high pathogenic significance. Based on the above, *ERBB3* is highly likely to be pathogenic. However, although the pathological significance of *CDKN2A* is unclear in the strict sense, it may be an important mutation in the development and maintenance of cancer. Because these 3 gene mutations are shared, the 2 tumors are unlikely to be collision tumors, and it is more likely that the 2 tumors originated from a common source.

Because a nonsense mutation in *TP53* was identified, it was predicted that a premature stop codon truncation mutation would result in null p53 expression (completely negative). However, neither tumor showed a null pattern. Instead, there were scattered cells that weakly expressed p53 in its wild-type form. Previous reports have shown that p53 staining is very useful for predicting *TP53* mutations, but not perfect.^19,20)^ To our knowledge, there have been no reports of p53 staining for the *TP53* c.1015G>T (p.Glu339Ter) mutation detected in this study.

There are 3 possible reasons why p53 immunostaining was positive in this study despite the presence of a *TP53* nonsense mutation: (1) the *TP53* c.1015G>T (p.Glu339Ter) mutation may be detected because it retains the epitope (peptides 35 to 45 near the N-terminus) recognized by the p53 antibody (DO-7, Nichirei Biosciences, Tokyo, Japan); (2) the C-terminus is missing, which may affect p53 ubiquitination, resulting in the truncated p53 remaining undegraded by the ubiquitin–proteasome system^21)^; and (3) the premature stop codon is close to the normal stop codon, which may allow protein production from abnormal mRNA by avoiding the mRNA degradation mechanism caused by the nonsense mutation (55-nt rule).^22)^ Overall, relatively rare *TP53* mutation patterns, in which p53 is neither overexpressed nor lost, may lead to the formation of MiNEN in adenocarcinomas and NET.

Further, with regard to the direction of differentiation, it is thought that the adenocarcinoma differentiated into NET. Immunostaining revealed that some of the adenocarcinoma cells in the transitional zone of the tumor were positive for NET markers (chromogranin A and synaptophysin) (**Fig. 4D** and **4F**). Thus, it is possible that the adenocarcinoma acquired neuroendocrine properties, or the NET lost these properties. On the other hand, macroscopic findings showed adenocarcinoma spreading to both sides of the NET. Instead of considering that the cells on both sides of the NET lost their NET properties, we assume that it was more likely that some of the extensive adenocarcinoma cells acquired neuroendocrine properties and formed a tumor.

In this case, a clone that differentiated into a more benign tumor from a highly malignant tumor survived and proliferated, but the exact mechanism is difficult to explain. Unknown genetic mutations that could not be elucidated in this study may be involved in its differentiation into a low-grade tumor. Ki-67 expression, which indicates proliferation, was present in 46% of the adenocarcinoma cells and 14% of the NET cells. Furthermore, NET cells had lower proliferation than adenocarcinoma cells. Although proliferation was indeed reduced, the 14% of NETs that expressed Ki-67 provides sufficient proliferative ability, so it is highly likely that they survived and proliferated.

This study has some limitations. WES data identified previously reported pathogenic variants in *TP53*, *ERBB3*, and *CDKN2A* as common somatic mutations. However, because different exon variants of unknown pathogenic significance were identified in the 2 histological types, it is possible that there may be unknown mutations among them that distinguish adenocarcinoma from NET. Copy number analysis, structural abnormalities, and the presence of fusion genes due to these abnormalities were not identified in this study. The results of this study suggest that the 2 tumors had a common origin. However, with only WES data, it is difficult to exactly explain the differences between adenocarcinomas and NETs. Performing whole-genome sequencing or RNA sequencing with normal tissue as a control would provide a more detailed understanding of the pathogenesis.

## CONCLUSIONS

In this case report, MiNEN with a NET component was shown to have a common origin, as each tumor component had a common genetic mutation. Furthermore, the NEN component of MiNEN occurring in the gallbladder was associated with a *TP53* mutation, despite the low frequency of *TP53* mutations in normal NETs.

## ACKNOWLEDGMENTS

We thank Yoko Yokoyama and Hiroko Matsuda (Education and Research Support Center at Gunma University) for their technical support in DNA extraction. We would also like to thank Editage (www.editage.com) for English language editing.

## DECLARATIONS

### Funding

No grant support or funding was received from public institutions or private enterprises.

### Authors’ contributions

TS reported this case and wrote the manuscript.

HS and KA were involved in treating the patient and helped draft the manuscript.

YM, KT, RK, TY, TS, and KH contributed to the analysis of gene expression and WES data.

YT and HI diagnosed the pathological findings.

KS participated in critically revising the manuscript.

All the authors have read and approved the final manuscript.

Accountability for all aspects of the work: all authors.

### Availability of data and materials

The datasets supporting the conclusions of this article are included within the article.

### Ethics approval and consent to participate

This study was approved by the ethical review board for medical research at Gunma University (approval number: HS2023-030) and complied with the principles of the Declaration of Helsinki.

### Consent for publication

Written informed consent was obtained from the patient for the publication of this case report and accompanying images.

### Competing interests

The authors declare that they have no competing interests.
